
JOURNAL INFORMATION
==============================
NLM Title Abbreviation: Innov Aging
Journal ID: Innov Aging
NLM Journal ID: 101703706
Journal ID: innovateage

Innovation in Aging

EISSN: 2399-5300
Publisher: Oxford University Press

ARTICLE INFORMATION
==============================
PMCID: PMC6845308
DOI: 10.1093/geroni/igz038.968
Article ID: igz038.968
Article version: 1
Subjects: Session 1315 (Poster), Chronic Disease and Disease Management

SOCIOEMOTIONAL SELECTIVITY THEORY AND THE PERCEPTION OF CHRONIC PAIN

Wagstaff Ruth A
Knight Bob G
Hendry Liam
University of Southern Queenslan , Toowoomba, Queensland, Australia
Publication date: 2019 Nov
Electronic publication date: 2019 Nov 8
Volume: 3
Issue: Suppl 1
Issue title: Program Abstracts from the GSA 2019 Annual Scientific Meeting “Strength in Age—Harnessing the Power of Networks”
First page: S259
Last page: S259
Copyright: © The Author(s) 2019. Published by Oxford University Press on behalf of The Gerontological Society of America.
Copyright year: 2019
License: This is an Open Access article distributed under the terms of the Creative Commons Attribution License (http://creativecommons.org/licenses/by/4.0/), which permits unrestricted reuse, distribution, and reproduction in any medium, provided the original work is properly cited.
License URL: https://creativecommons.org/licenses/by/4.0/

Page count: 1

==============================
### SOCIOEMOTIONAL SELECTIVITY THEORY AND THE PERCEPTION OF CHRONIC PAIN

Ruth A. Wagstaff,[1] Bob G. Knight,[1] and Liam Hendry[1],
*1. University of Southern Queenslan, Toowoomba, Queensland, Australia*

The Fear Avoidance model of chronic pain (FAM) posits that pain perception is best understood in the context in which pain occurs. An aspect of context that has received little attention is the effect that future time perspective and its related goals may have on chronic pain perception. According the Socioemotional Selectivity Theory (SST), young adults have instrumental goals to prepare for the future and older adults have goals to establish and maintain wellbeing. These differences have profound effect on cognition including attentional bias. It was hypothesised that future time perspective would influence pain perception through attention to pain. A total of 306 participants, 18 – 88 year olds with chronic joint pain completed the Numeric Analogue Scale for pain, three scales to measure the FAM constructs: Pain Vigilance Awareness Questionnaire, Pain Catastrophization Scale, and the Pain Anxiety Symptoms Scales, and, two scales to measure the SST constructs: Future Time Perspective Scale, (measures the goals and opportunities anticipated in the remaining life span) and the Positive Affect Negative Affect Scale (measures mood). Structural equation modelling finding supported the hypothesis and the model of fit indices indicated a good fit ($\chi 2$ (53) = 127.412, p = .000, TLI .958, CFI = .971, SRMSEA = .056, RMSEA = .068, CI 90% [.053, .083]). This is the first time that age, future time perspective and the positivity effect have been shown to contribute the pain experience through attention. It provides a possible framework for exploring age appropriate psychological treatments for chronic pain.
